# Population-based human immunodeficiency virus 1 drug resistance profiles among individuals who experienced virological failure to first-line antiretroviral therapy in Henan, China during 2010–2011

**DOI:** 10.1186/s12981-015-0062-y

**Published:** 2015-06-27

**Authors:** Jia Liu, Yasong Wu, Wenjie Yang, Xiujuan Xue, Guoqing Sun, Chunhua Liu, Suian Tian, Dingyong Sun, Qian Zhu, Zhe Wang

**Affiliations:** Henan Center for Disease Control and Prevention, Zhengzhou, Henan China; Chinese Center for Disease Control and Prevention, Beijing, China

**Keywords:** HIV, Drug resistance, First-line antiretroviral therapy, Virological failure

## Abstract

**Background:**

In Henan, China, first-line antiretroviral treatment (ART) was implemented early in a large number of treatment-experienced patients who were more likely to have a drug resistance. Therefore, we investigated the human immunodeficiency virus (HIV)-1 drug resistance profiles among patients in Henan who experienced virological failure to ART.

**Method:**

A cross-sectional survey was administered in 10 major epidemic cities from May 2010 to October 2011. Adult patients who experienced virological failure (virus load ≥1,000 copies/mL) with >1 year of first-line antiretroviral treatment consented to provide blood for genotype resistance testing. The clinical and demographic data were obtained from the patients’ medical records. Logistic regression analysis was performed to determine the factors associated with ≥1 significant drug resistance mutation.

**Results:**

We included 3,235 patients with integral information and valid genotypic resistance data. The city, age, CD4 counts, virus load, treatment duration, and World Health Organization stage were associated with drug resistance, and 64.76% of patients acquired drug resistance. The nucleoside reverse transcriptase inhibitor (NRTI), non-(N)NRTI, and protease inhibitor resistance mutations were found in 50.26, 63.12, and 1.30% of subjects, respectively. Thymidine analogue mutations, NNRTI and even multidrug resistance complex were quite common in this patient cohort.

**Conclusion:**

Multiple and complex patterns of HIV-1 drug resistance mutations were identified among individuals who experienced virological failure to first-line ART in Henan, China during 2010–2011. Therefore, timely virological monitoring, therapy adjustments, and more varieties of drugs and individualized treatment should be immediately considered in this patient population.

## Background

Continuous improved antiretroviral therapy (ART) has been one of the most significant breakthroughs in human immunodeficiency virus (HIV)/acquired immune deficiency syndrome (AIDS) prevention and control over the past 30 years because of its outstanding effect on reducing AIDS mortality and morbidity [1, 2], ameliorating quality of life, and preventing HIV-1 infection [3]. However, the emergence of HIV drug resistance is inevitable following the large-scale rollout of ART. The enhanced resistance to first-line antiretroviral drugs has led to persistent viral replication that impairs patients’ immunological and clinical status, and the accumulation of resistance mutations after continuing a failing regimen may compromise the second-line therapy [4]. To maximize the long-term effectiveness of first-line ART regimens and to ensure the sustainability of ART programs, it is essential to minimize the further spread of HIV drug resistance. The World Health Organization (WHO) recommends that the scale-up of HIV ART should always be accompanied by a robust assessment of drug resistance emergence [5].

The Chinese government initiated the China Comprehensive AIDS Response program to provide free HIV treatment in 2002, which rapidly scaled up the National Free Antiretroviral Therapy Program. Subsequently, drug resistance surveillance in different areas was attempted, and the popularization of drug resistance monitoring was performed in 2010. The Henan Province has always drawn concern because of the extensive spread of HIV among former plasma donors and the earlier initiation of the National Free ART Program with a large number of ART patients [6, 7]. Numerous HIV drug resistance studies with heterogeneous designs, defined areas, and methods have been performed in China with small populations from Henan [8–11]; however, until recently, few studies have illuminated the typical prevalence and pattern of HIV drug resistance in the Henan Province. Therefore, we assessed the HIV-1 drug resistance profile among first-line ART patients in Henan who experienced virological failure.

## Method

### Study design

The administration of ART, delivery of antiviral drugs, adherence education, and collection of plasma specimens mainly depended on the local centers for disease control and prevention (CDC) and the community clinics in the Henan Province. The CD4 cell counts were measured twice and virus load were measured once 1 year by a local CDC and Henan CDC, and the results were provided to treating physicians and the national HIV/AIDS case reporting system. All the cases of HIV/AIDS in China are reported via the national HIV/AIDS case reporting system. Records in this system include identity and demographic information, HIV testing results, the self-reported transmission route, and health status information. Base data for this study was derived from this system. For the purpose of this study, virological failure patients (defined as having an HIV virus load of ≥1,000 copies/mL) underwent genotypic drug resistance testing and the results were also provided to treating physicians and the national HIV/AIDS case reporting system.

### Ethics statement

Written consent was obtained from patients to participate in the China’s routine HIV/AIDS surveillance program and the regular administration of the national ART program, and parental consent was also obtained on behalf of the participants aged less than 18 years. Since the data and the samples used in this study were collected as a function of surveillance program above, no additional informed consent was required. This study was reviewed and approved by the Institutional Review Board of the Henan CDC.

### Participants

According to the aforementioned conditions, first-line ART-virological failure patients (aged ≥15 years) were selected if the virus load was ≥1,000 copies/mL with >1 year of treatment from May 2010 to October 2011 in 10 cities that held the highest HIV-1 epidemic in the Henan Province. The patients could not followed or refused to give samples were not included. The first-line ART regimens consisted of two nucleoside reverse transcriptase inhibitors (NRTIs): (azidothymidine [AZT] or stavudine [D4T]) + (didanosine [DDI] or lamivudine [3TC]) and one non-(N)NRTIs: (nevirapine [NVP] or efavirenz [EFV]). AZT, D4T, DDI, and NVP are generically produced in China, whereas 3TC and EFV are branded drugs that became available in 2005.

### Laboratory tests

The CD4 cell counts were measured using flow cytometry by the local CDCs. The plasma HIV-1 virus load was tested by the local city and Henan CDCs using the Abbott Real-Time HIV-1 assay (Abbott Molecular Inc., Des Plaines, IL, USA), NucliSENS EasyQ-EasyMag HIV-1 assay (bioMerieux, Marcy l’Etoile, France), or Cobas AmpliPrep-Cobas TaqMan HIV-1 assay (Roche Diagnostics, Mannheim, Germany). Samples with a virus load of ≥1,000 copies/mL underwent HIV-1 drug resistance genotyping using an in-house method developed by the national core laboratories as described in previous studies [12, 13]. A fragment of the HIV-1 pol gene (protease, amino acids 1–99, and part of the reverse transcriptase amino acids 1–252) was bulk-sequenced from the plasma ribonucleic acid (RNA). The resulting fragment was analyzed for subtype, drug resistance mutations and drug susceptibility using the Stanford HIV Drug Resistance Database (http://hivdb.stanford.edu).

### Data analysis

The demographic variables were analyzed using descriptive statistics, and the risk factors for developing HIV drug resistance were analyzed using logistic regression. Variables associated with resistance in the univariate analyses (*p* value <0.05) and those clinically meaningful were included in the multivariable regression model. The tests were two-sided, and a p-value <0.05 indicated statistical significance. All the statistical analyses were performed using SPSS, version 19.0 (IBM Corp., Armonk, NY, USA).

## Result

### Demographic characteristics

**Figure 1:**
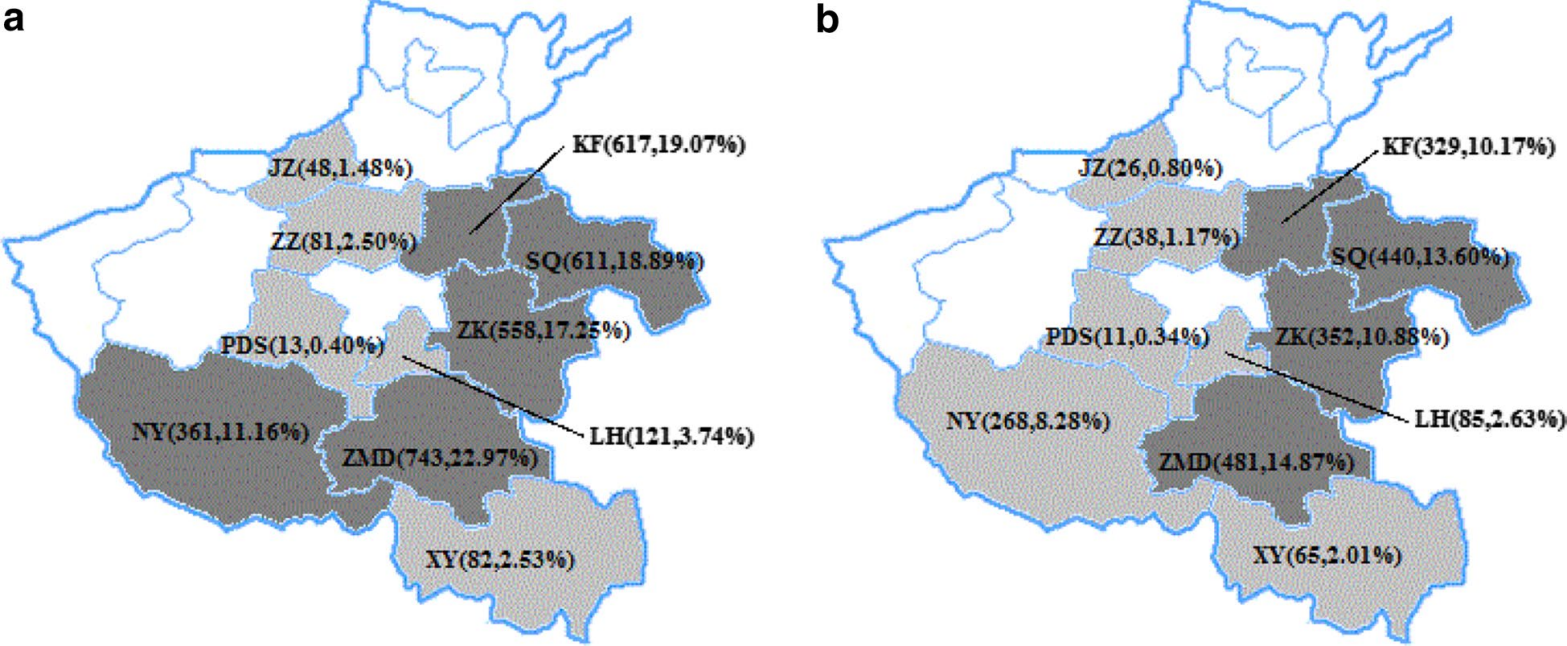
The geographical distribution of subjects according to the cities analyzed in this study. **a** The geographical distribution of treatment failures. **b** The geographical distribution of drug resistance genotypes detected. The abbreviation of city names are ZMD (Zhumadian), ZZ(Zhengzhou), KF(Kaifeng), JZ(Jiaozuo), ZK(Zhoukou), PDS(Pingdingshan), LH(Luohe), SQ(Shangqiu), NY(Nanyang), XY(Xinyang). The *number* and *percentage* in the brackets are the absolute number of subjects and the percent in the 3235 individuals. *Heavy gray*, cities with >10% of subjects; *light gray*, cities with <10% of subjects.

In our study, 4,020 individuals were investigated and sampled, which included 23 non-Henan, 154 dual-tested. Moreover, 81 patients with incomplete information and 527 with a fragmentary sequence were excluded, leaving 3,235 subjects in the final analysis (Table 1). The following five cities accounted for a substantially larger proportion: Zhumadian (22.97%), Kaifeng (19.07%), Shangqiu (18.89%), Zhoukou (17.25%), and Nanyang (11.16%) (Figure 1). Of the 3,235 patients, 63.37% were aged 31–50 years, 52.49% were male, 82.87% were married, 90.11% reported former plasma donors as the route of HIV infection, 39.54% had WHO stage II HIV disease, 51.56% had been treated for >6 years, 27.02% have CD4 counts ≥350 cells/μL, 58.83% have virus load in between 3 and 4 log copies/ml. All the 3235 individuals were B subtypes. ART was initialized with AZT/D4T + DDI + NVP/EFV in 1,428 subjects (44.14%), with AZT/D4T + 3TC + NVP/EFV in 1,170 (36.17%). The regimen that included DDI was substituted with a regimen that included 3TC after 2005 when it was widely available.

**Table 1:** The subjects’ characteristic and the factors associated with at least one human immunodeficiency virus (HIV) drug resistance mutation

Variable	Number (%)	HIV drug resistance (%)	Crude OR (95% CI)	P-value	Adjusted OR (95% CI)	P-value
City						
Zhumadian	743 (22.97)	481 (64.74)				
Zhengzhou	81 (2.50)	38 (46.91)	0.5 (0.3,0.8)	0.00	0.8 (0.5,1.4)	0.49
Kaifeng	617 (19.07)	329 (53.32)	0.6 (0.5,0.8)	0.00	0.7 (0.6,0.9)	0.01
Jiaozuo	48 (1.48)	26 (54.17)	0.6 (0.4,1.2)	0.14	0.8 (0.4,1.6)	0.59
Zhoukou	558 (17.25)	352 (63.08)	0.9 (0.7,1.2)	0.54	1.1 (0.9,1.5)	0.33
Pingdingshan	13 (0.40)	11 (83.33)	3.0 (0.7,13.6)	0.16	3.1 (0.7,14.8)	0.15
Luohe	121 (3.74)	85 (70.25)	1.3 (0.8,2.0)	0.24	1.7 (1.1,2.8)	0.02
Shangqiu	611 (18.89)	440 (72.01)	1.4 (1.1,1.8)	0.00	1.5 (1.2,1.9)	0.00
Nanyang	361 (11.16)	268 (74.24)	1.6 (1.2,2.1)	0.00	1.9 (1.4,2.5)	0.00
Xinyang	82 (2.53)	65 (79.27)	2.1 (1.2,3.6)	0.01	2.4 (1.3,4.4)	0.00
Age						
≤30	89 (2.75)	70 (78.65)				
31–50	2,050 (63.37)	1,295 (63.17)	0.5 (0.3,0.8)	0.00	0.5 (0.2,0.8)	0.01
>50	1,096 (33.88)	730 (66.61)	0.5 (0.3,0.9)	0.02	0.5 (0.3,0.9)	0.03
Gender						
Female	1,537 (47.51)	1,011 (65.78)				
Male	1,698 (52.49)	1,084 (63.84)	0.9 (0.8,1.1)	0.25		
Married						
No	2,681 (82.87)	1,737 (64.79)				
Yes	554 (17.13)	358 (64.62)	1.0 (0.8,1.2)	0.94		
HIV transmission route						
FPD	2,915 (90.11)	1,909 (65.49)				
Sexual intercourse	238 (7.36)	139 (58.40)	0.7 (0.6,1.0)	0.03	1.1 (0.8,1.5)	0.71
Mother-to-Child	24 (0.74)	22 (91.67)	5.8 (1.4,24.7)	0.02	3.3 (0.7,16.6)	0.14
Other	58 (1.79)	25 (43.10)	0.4 (0.2,0.7)	0.00	0.8 (0.4,1.4)	0.36
CD4						
≥350	874 (27.02)	536 (61.33)				
200–349	1,112 (34.37)	718 (64.57)	0.9 (0.7,1.1)	0.20	0.9 (0.7,1.2)	0.52
50–199	1,011 (31.25)	672 (66.47)	1.3 (1.0,1.7)	0.03	1.4 (1.1,1.9)	0.01
<50	238 (7.36)	169 (71.01)	1.9 (1.3,2.7)	0.00	2.6 (1.7,4.0)	0.00
Virus load						
>4log	1,332 (41.47)	811 (60.89)				
3–4log	1,903 (58.83)	1,284 (67.47)	1.4 (1.1,1.9)	0.01	1.6 (1.1,2.0)	0.01
Initial ART regimen						
AZT/D4T + DDI + EFV/NVP	1,428 (44.14)	975 (68.28)				
AZT/D4T + 3TC + EFV/NVP	1,170 (36.17)	714 (61.03)	0.7 (0.6,0.9)	0.00	1.0 (0.8,1.2)	0.85
Other regimens	637 (19.69)	406 (63.74)	0.8 (0.7,1.0)	0.04	0.9 (0.8,1.2)	0.61
WHO stage						
I	635 (19.63)	382 (60.16)				
II	1,279 (39.54)	829 (64.82)	1.2 (1.0,1.5)	0.05	1.1 (0.9,1.4)	0.32
III	860 (26.58)	573 (66.63)	1.3 (1.1,1.6)	0.01	1.4 (1.0,1.7)	0.01
IV	226 (6.99)	146 (64.60)	1.2 (0.9,1.7)	0.24	1.4 (1.0,1.9)	0.08
No information	235 (7.26)	165 (70.21)	1.6 (1.1,2.2)	0.01	0.9 (0.6,1.3)	0.63
Duration of ART (year)						
7–	930 (28.75)	650 (70.49)				
6–	738 (22.81)	502 (68.02)	0.9 (0.7,1.1)	0.41	0.9 (0.7,1.1)	0.24
5–	331 (10.23)	241 (72.81)	1.2 (0.9,1.5)	0.32	0.9 (0.7,1.2)	0.60
4–	264 (8.16)	182 (68.94)	1.0 (0.7,1.3)	0.77	0.7 (0.5,1.0)	0.05
3–	253 (7.82)	171 (67.59)	0.9 (0.7,1.2)	0.48	0.6 (0.4,0.9)	0.00
2–	289 (8.93)	183 (63.32)	0.7 (0.6,1.0)	0.04	0.5 (0.4,0.7)	0.00
1–	430 (13.29)	166 (38.60)	0.3 (0.2,0.3)	0.00	0.2 (0.1,0.2)	0.00

### Risk factors associated with genotypic human immunodeficiency virus drug resistance

Exploratory logistic regression analysis was performed to assess the factors that were significantly associated with resistance (Table 1). According to the univariate logistic regression model, eight potential factors correlated with HIV drug resistance. In the multivariate model, the following six factors had correlations: the city (patients in Xinyang had the highest level of drug resistances, 79.27%); age (patients in <30 years group had the highest level of drug resistance, 78.65%); CD4 counts (patients in <50 cells/μL group had the highest level of drug resistance, 71.01%); Virus load (patients in 3-4 log copies/ml group had the highest level of drug resistance, 67.47%); WHO stage (patients in WHO stage III group had the highest level of drug resistance, 66.63%); and treatment duration (patients in 5–6 years group had the highest level of drug resistance, 72.81%).

### Pattern and frequency of predicted HIV drug resistance mutations

**Figure 2:**
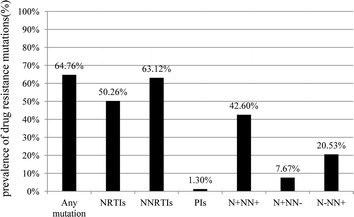
The prevalence of predicted human immunodeficiency virus 1 drug resistance mutations in different drug classes among individuals experiencing virological failure to first-line antiretroviral treatment in Henan, China during 2010–2011. *N+NN+* the existence of both nucleoside reverse transcriptase inhibitor (NRTI) and non-(N)NRTI resistance mutation, *N+NN*− the existence of NRTI but not NNRTI resistance mutations, *N-NN+* the existence of NNRTI but not NRTI resistance mutations.

At least one significant HIV drug resistance mutation was identified in 2,095 (64.76%) patients, according to the Stanford HIV Drug Resistance Database. The frequency of NNRTI, NRTI, and PI resistance mutations were 63.12, 50.26, and 1.30%, respectively. In this study, 42.60% of patients had both NRTI and NNRTI resistance mutations, 7.67% had NRTI but not NNRTI, and 20.53% had NNRTI but not NRTI resistance mutations (Figure 2).

M184 V/I (35.64%) emerged as the most common NRTI resistance mutation, followed by thymidine analogue mutations (TAMs): T215Y/F (27.17%), M41L (18.39%), D67 N (12.21%), K70R/E/G (10.20%), L210 W (10.17%), and K219E/Q (10.02%). The prevalence of >1 TAMs was 42.32%. Furthermore, the prevalence of M184 V/I + TAMs was 21.36%, TAMs-1 (M41L, L210 W, and T215Y) was 8.96%, and TAMs-2 (D67 N, K70R, T215F, and K219Q) was 4.61%. Fifty-one (1.58%) and thirteen (0.40%) patients had the K65R/N and Q151 M complex, respectively. Among the NNRTI resistance mutations, K103 N (34.84%) was the most common NNRTI, followed by Y181C/V/I (22.04%) and G190A/S/E (18.24%) (Table 2).

**Table 2:** Frequency of the predicted human immunodeficiency virus drug resistance mutations

Resistance mutation (n = 3,235)	No. of patients (%)
NRTI mutation	
M184V/I	1,153 (35.64)
T215Y/F	879 (27.17)
M41L	595 (18.39)
D67N	395 (12.21)
K70R/E/G	330 (10.20)
L210 W	329 (10.17)
K219E/Q	324 (10.02)
L74I/V	118 (3.65)
V75I/M	110 (3.40)
Q151 M	93 (2.87)
A62 V	74 (2.29)
F116Y	67 (2.07)
K65R/N	51 (1.58)
F77L	30 (0.93)
Y115F	9 (0.28)
≥1TAM	1,369 (42.32)
TAM-1	290 (8.96)
TAM-2	149 (4.61)
TAM-1/TAM-2	10 (0.31)
Q151 M complex	13 (0.40)
M184 V/I + TAMs	691 (21.36)
NNRTI mutation	
K103 N/S	1,127 (34.84)
Y181C/V/I	713 (22.04)
G190A/S/E	590 (18.24)
K101E/P	247 (7.64)
V108I	225 (6.96)
V106A/M	177 (5.47)
Y188L/C/H	140 (4.33)
F227L/C	121 (3.74)
K238T	117 (3.62)
V179D/E/F	93 (2.87)
P225H	63 (1.95)
A98G	53 (1.64)
L100I	11 (0.34)
P236L	1 (0.03)
PI mutation	
M46I/L	22 (0.68)
V82F	11 (0.34)

### Estimated susceptibility to various antiretroviral drugs

**Figure 3:**
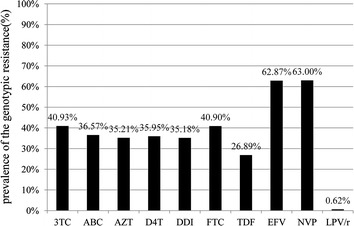
Prevalence of the genotypic resistance against different antiretroviral treatment (ART) drugs among individuals experiencing virological failure to first-line ART in Henan, China during 2010–2011.

Mutational patterns were used to estimate the drug susceptibility in patients based on the Stanford University HIV Drug Resistance Database (Figure 3). Of the 40.93 and 40.90% subjects in this cohort had an estimated resistance against 3TC and FTC; 35–37% against ABC, AZT, D4T, and DDI; and 26.89% against TDF. There was a comparatively high prevalence of an estimated resistance to EFV and NVP, which reached 62–63%, and 0.62% of the subjects had a resistance to lopinavir.

## Discussion

ART has expanded in Henan during the last decade, and the continued success of ART programs will require an understanding of the profiles of HIV drug resistance among individuals in whom the treatment has failed. This study’s findings demonstrated the presence of HIV drug resistance among individuals from the Henan Province of China (2010–2011) in whom the first-line therapy had virologically failed.

In the early years, the magnitude of the focus was on former plasma donors, however, with the change of the HIV epidemic, sexual intercourse has been the focus of HIV/AIDS control and prevention, which has attracted substantial resources [14–17]. This study had a large number of subjects, and 90.11% of the virological failure population accounted for former plasma donors. This finding indicates that these people also currently occupy an important position in the ART programs; thus, measurements such as virology surveillance, drug resistance monitoring, therapy adjustments, and related social security need to be ensured.

The prevalence of HIV drug resistance is not constant across Henan. Zhumadian, Kaifeng, Shangqiu, Nanyang, and Xinyang have assembled a great deal of resources in the field of HIV/AIDS control and prevention because of their high HIV epidemic [14]. Compared to Zhumadian, multivariate analysis showed that Shangqiu, Nanyang, and Xinyang were associated with a higher likelihood of drug resistance mutations, whereas Kaifeng was not. Similarly, the other five cities also have the disconcordance phenomenon. These findings clarified that the scale of epidemic and resource focus on HIV/AIDS control and prevention has no direct correlation with the development of HIV drug resistance; however like other study had set forth, the local ART programmatic factors play a significant role [10]. Patients with low CD4 counts (below 200 cells/μL) and long-time treatment(over 4-years) were at the high risk of HIV drug resistance in this study that can be easily understood. The lower virus load(3–4 log copies/ml other than >4 log copies/ml) were correlated to the high risk of HIV drug resistance mainly because of the HIV drug resistance strains were defective virus, and it probably have a lower capability of replication [4, 18].

NNRTIs have a low genetic barrier to resistance. Therefore, a single mutation is often sufficient for causing resistance to the currently recommended first generation of NNRTIs, NVP, or EFV [19, 20], which is why the second-line therapy did not include NNRTIs. Henan initiated the switch to second-line ART in patients who experienced failure with the first-line treatment in 2009. In this study, the prevalence of NNRTI resistance mutations (63.12%) was approximately equal to the total resistance mutations (64.76%). In addition, 20.53% of the patients had the NNRTI resistance mutations without NRTI, while only 7.67% had NRTI without NNRTI. Lastly, the prevalence of estimated resistance against EFV (62.87%) and NVP (63.00%) was both high with no significant different. All of these findings illustrated that the NNRTI resistance was more pervading than NRTI in ART failure patients. Therefore, it is just and reasonable for the government to perform second-line ART.

As expected, M184 V/I was the most commonly detected NRTI resistance mutation, which was also found in previous studies [21, 22], and this was followed by a high frequency of TAMs. A comparative amount of TAMs-1 and TAMs-2 were also detected in this study. TAMs confer cross-resistance to AZT and D4T as well as to most NRTIs in varying degrees when enough TAMs have accumulated. Additionally, two typical TAM pathways with a high resistance to all NRTIs were discovered: TAM-1 (M41L, L210W, and T215Y) and TAM-2 (D67N, K70R, K219E/Q, and T215F) [23–25]. Fewer K65R and Q151M complexes were detected in the study, mainly because they were apt to other subtypes and regimens [26, 27]. The Q151M complexes and K65R were also resistance mutations to multiple or even all of the NRTIs. The Q151M complex confers resistance to all NRTIs, except for TDF [28], and K65R confers resistance to D4T and TDF and possibly also to 3TC/FTC, DDI, and ABC [29]. Among patients with a detectable HIV RNA at 12 months, the HIV drug resistance was primarily due to M184V and NNRTI mutations. Complex mutation patterns, including the TAMs, K65R, and multinucleoside mutations, are prevalent among cases of treatment failure that were identified by clinical or immunologic methods as a result of the accumulation of drug resistance [21, 22]. The accumulation of drug resistance depends on the frequency of monitoring [30] and belated regimen adjustment. Thus, the duration of virologic failure must be somewhat longer than it should be because of the delayed virology surveillance or therapy adjustment in Henan during 2010–2011. Meanwhile, as Figure 3 shows, 35–40% of patients showed an estimated resistance against most NRTIs with 40.39 and 26.89% against 3TC and TDF, respectively. Since the second-line ART consists of two NRTIs (usually 3TC and TDF) and one PI, all of these findings suggest that a considerable amount of patients in Henan may already be compromised to second-line ART, indicating that approaches to more newer drugs with different mechanisms and individualized treatment should be considered sooner.

The emergence of transmitted HIV drug resistance can be anticipated, because such a large quantity of individuals acquired HIV drug resistance in this population-based survey. Recently, a moderate level of prevalence for transmitted HIV drug resistance has been reported in Henan [31]. More active monitoring strategies, such as conducting drug resistance testing prior to the initiation of ART should be performed as recommended by WHO in developed countries [32, 33].

Drug resistance mutations were undetected in 35.26% of patients among this treatment failure cohort, a significant proportion of individuals without mutations for treatment failure has also been reported in all similarity studies [5]. This probably because HIV drug resistance presented in some patients as minority variants (lower than 10–20% of the virus population) that may pass undetected by Sanger sequencing method [18], and non-adherence or treatment interruption must be another etiology of treatment failure without drug resistance. This study has gaps in knowledge on adherence, and we were not able to determine the duration of virological failure by using a cross-sectional survey. In view of these limitations, a further study needs to be conducted to provide sufficient evidence for evaluating a more precise and comprehensive profile of drug resistance among ART failures in Henan.

## Conclusion

In conclusion, long-term ART roll-out leads to a comparatively high prevalence of HIV drug resistance among individuals who have experienced virological failure to first-line ART in Henan during 2010–2011, indicating a magnitude of former plasma donors. Multiple mutations and complex patterns have been identified, which may cause a growing number of those being treated with second-line therapy with partial or no activity. Performing routine drug resistance surveillance programs and timely therapy adjustment, especially among former plasma donors, ensuring access to new kinds of drugs, and initiating individualized treatment must be a priority in order to preserve programmatic treatment success in long-life therapy.

*Data and materials availability* Sequences generated for this study were deposited in the national AIDS treatment database of China.
